# Enhancing the Biological Effects of Bioactive Compounds from Microalgae through Advanced Processing Techniques: Pioneering Ingredients for Next-Generation Food Production

**DOI:** 10.3390/foods13121811

**Published:** 2024-06-08

**Authors:** Monize Bürck, Sergiana dos Passos Ramos, Anna Rafaela Cavalcante Braga

**Affiliations:** 1 Postgraduation Program in Nutrition, Universidade Federal de São Paulo (UNIFESP), São Paulo 04023-900, SP, Brazil; monize.burck@unifesp.br; 2 Nutrition and Food Service Research Center, Universidade Federal de São Paulo (UNIFESP), Santos 11015-020, SP, Brazil; 3 Department of Biosciences, Universidade Federal de São Paulo (UNIFESP), Santos 11015-020, SP, Brazil; sergiana.passos@unifesp.br; 4 Department of Chemical Engineering, Universidade Federal de São Paulo (UNIFESP), Diadema 04021-001, SP, Brazil

**Keywords:** innovative applications, algae, sustainable, food processing

## Abstract

The heightened interest in healthy dietary practices and the preference for fresh, minimally processed foods with reduced additives have witnessed a significant surge among consumers. Within this context, bioactive compounds have garnered attention as potent agents offering beneficial biological effects when integrated into food formulations. Nevertheless, the efficacy of these bioactive compounds in product development encounters numerous challenges during various processing and storage stages due to their inherent instability. Addressing these limitations necessitates exploring novel technological approaches tailored explicitly to the application of bioactive compounds in food production. These approaches should not only focus on preserving the bioactive compounds within food matrices but also on retaining the sensory attributes (color, taste, and aroma) of the final food products. The impact of microalgae and their bioactive compounds on human health and well-being has been extensively reported in the literature. However, there is still a gap regarding the processing and stability of microalgal bioactive compounds to improve their application in the food industry. The main goal of the present work is to point out how to overcome technological challenges in enhancing the stability of bioactive compounds from microalgae for optimal food applications.

## 1. Introduction

The world’s population is currently around 8 billion people and is projected to increase to around 9.7 billion by 2050. This growth highlights the need to produce more food, which requires significant land and resources. However, this unsustainable approach leads to environmental degradation and resource depletion. Researchers are exploring alternative food sources to address the future demand for ingredients, particularly protein [1].

One promising alternative is microalgae, which offer advantages like rapid growth, a high protein content, a rich amino acid profile, and several bioactive compounds, including natural pigments. Microalgae encompass various types in different environments and can be unicellular or multicellular organisms [2,3,4,5]. 

The global market for microalgae is growing, and certain species, like *Limnospira* (previously known as *Arthrospira* and commonly known as Spirulina) and *Chlorella*, are the most studied microalgae. They contain high levels of protein and offer nutritional benefits. Microalgal farming presents an eco-friendly solution as it can utilize carbon dioxide and other emissions as nutrients. Additionally, microalgae are adaptable to various environments and are generally considered safe for human consumption [6]. Commercially, microalgal production is a goldmine, promising abundant biomass, CO_2_ absorption, and wastewater treatment benefits. While the global economy faced challenges in 2020, the microalgal industry thrived, hinting at a future worth of one billion by 2027 [7]. 

The interest in microalgae as a food ingredient source has increased due to their sustainability, nutritional value, and environmental benefits [3,8]. Research in this area is rapidly expanding, showing the potential of microalgae as a valuable food source for the future. Diving into the world of microalgae reveals a universe of wonders. These photosynthetic powerhouses, ranging from microns to hundreds of microns, are more than just small beings; they are versatile, adaptable, and full of bioactive potential: a treasure trove of bioactive compounds, including a range of natural pigments such as chlorophylls, carotenoids, phycobiliproteins, and phenolic compounds, among others [9], providing exceptional biological properties as antioxidant, anticancer, antidiabetic, and anti-inflammatory properties [10,11]. 

On the other hand, challenges in utilizing bioactive compounds in industries arise from issues like low stability during processing and storage. These factors impact their effectiveness and can lead to the degradation of final products. 

To address this, promising future trends involve several processing techniques, including incorporating bioactive compounds into nanostructures [12], using high-pressure methods and a combination of ingredients, and developing new delivery systems (emulsions, nanoemulsions, gel, and bigels), as well as 3D printing, to overcome these hurdles and enhance product safety and quality at scale. 

The novelty of this review lies in its focus on utilizing advanced processing techniques to enhance the biological effects of bioactive compounds from microalgae. By doing so, this research aims to pioneer the development of innovative ingredients that can potentially revolutionize next-generation food production. This approach emphasizes the importance of bioactive compounds derived from microalgae. It underscores the significance of leveraging cutting-edge processing methods to maximize their benefits and applications in the food industry. Ultimately, this research contributes to expanding the frontiers of food science and technology by exploring new avenues for enhancing nutritional value, sustainability, and health-promoting properties in food products.

## 2. Microalgal Ingredients for Food Application

**Figure 1:**
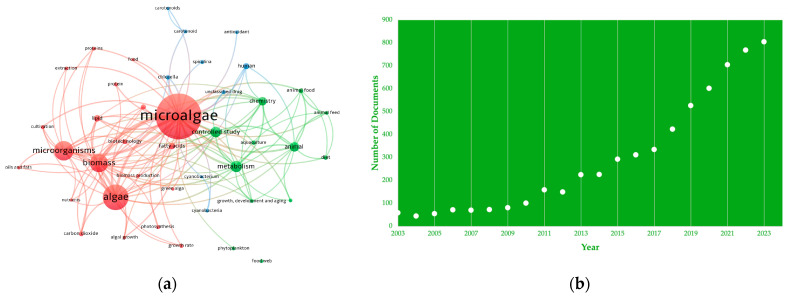
(**a**) Density visualization map resulting from the keywords applied in the search conducted in the present work using VosViewer software (VOSviewer version 1.6.20). (**b**) Published documents by year shown on the Scopus database.

A search using the Scopus database showed the most frequent keywords used for indexation (Figure 1a) and, in numbers, the increasing interest in microalgae as a food ingredient (Figure 1b). 

From the search, it was possible to notice that microalgae’s vast potential is being uncovered through ongoing exploration. Research efforts have delved into utilizing microalgae for additives, health supplements, and animal feed. Some microalgal varieties, like *Chlorella vulgaris*, *Euglena gracilis*, and *Nannochloropsis oceanica* CASA CC201, have gained approval as edible options in certain countries. Companies like VIVA Naturals have introduced products made from microalgae, such as GreenTrio Tanletten with Spirulina and Chlorella, offering benefits for digestive health. Despite these advancements, the widespread promotion and consumer adoption of microalga-based products are still in their early stages, facing various challenges that must be addressed [13]. 

Microalgal production holds considerable promise in sustainability and versatility, offering a renewable source of bioactive compounds and potential food ingredients. In terms of cultivation, microalgae’s high productivity and efficiency compared to traditional crops are well known. These microscopic organisms require minimal land, water, and resources to grow, which makes them environmentally sustainable. They can be cultivated in various environments, including freshwater, seawater, and wastewater, reducing the strain on arable land and freshwater resources [3,14].

Additionally, microalgae can uptake carbon dioxide and other nutrients from their surroundings, which makes them effective in bioremediation and nutrient recycling. They can be used in wastewater treatment plants to remove pollutants and excess nutrients, contributing to environmental sustainability [3,15]. The production costs of microalgae can vary depending on several factors, including the scale of cultivation, the type of microalgal species, cultivation methods, and downstream processing techniques. Initial investments in infrastructure, equipment, and research can be significant but may lead to cost savings in the long run due to microalgae’s high productivity and versatility. Still, the market prices of microalga-derived products can also vary based on purity, quality, production volume, and demand. Bioactive compounds extracted from microalgae are often priced at a premium due to their health benefits and unique properties. As technology advances and production processes become more efficient, the prices of microalga-based products are expected to become more competitive and accessible to a broader consumer base. Microalgal production offers a sustainable and economically viable solution for cultivating bioactive compounds with diverse applications in various industries, paving the way for innovative and eco-friendly products [16,17].

Research on the nutrient content of microalgae provides valuable and widely applicable insights into fundamental nutrients, such as carbohydrates, fats, proteins, minerals, vitamins, and dietary fiber. Carbohydrates are vital in microalgal cell structure, energy supply, and cellular regulation. Microalgal strains like *Pseudoneochloris*, *Scenedesmus*, and *Chlorella* are noted for their high carbohydrate content. Some microalgae, such as *Dunaliella* and *Klebsormidium*, excel in starch production, with impressive percentages relative to their dry weight. Additionally, dietary fiber, a crucial carbohydrate type, aids digestion and waste elimination. Fat is a vital component of human tissue and is a primary source of warmth for physiological functions. Microalgae boast abundant high-quality lipids that are recognized as valuable energy sources. In addition, protein is crucial in sustaining normal physiological functions, regulating bodily processes, and providing energy. Inadequate protein consumption can result in malnutrition [1,13,18].

Jadhav [19] summarized functional triacylglycerols from microalgae and their use in the formulation of functional foods, and showed that microalgae are rich sources of triacylglycerols with essential fatty acids and that they are being explored as potential alternatives to fish oil, catering to both functional ingredient needs and vegan preferences. Microalgal species such as *Crypthecodinium cohnii*, *Phaeodactylum tricornutum*, and *Schizochytrium* sp. can produce high polyunsaturated fatty acids (PUFAs). These functional triacylglycerols from microalgae, which are rich in omega-3 and omega-6 fatty acids, offer health benefits and may help prevent non-communicable diseases. However, the availability of functional food products containing these microalga-derived triacylglycerols currently needs to be improved in the market.

All macronutrients cited can be added as food ingredients. Particular attention has been paid to protein from microalgae in recent years, mainly since some microalgal species showcased protein accumulation of up to 70% in their dry matter, rapid growth compared to terrestrial plants, and superior protein productivity per area when compared to crops like soybean, legumes, or wheat, which once again emphasizes their potential to emerge as a sustainable protein source in the future [2]. Moreover, proteins from microalgae exhibit favorable techno-functional characteristics, serving as effective agents for foaming, gelling, and emulsifying purposes. Furthermore, essential amino acids in microalgae, such as Spirulina, elevate their status as a valuable protein source, particularly beneficial for individuals adhering to vegetarian or vegan diets [20,21]. 

It is important to acknowledge that the amino acid profile in microalgae can differ based on factors like sources, cultivation techniques, and processing methods. For example, despite *Phaeodactylum tricornutum* having a lower protein content (43.5 g/100 g) [22] than *Liminospira* (57.5 g/100 g) [23], its protein content is comparable to loin beef (27.7 g/100 g) [24], which has sparked interest in this species. *P. tricornutum* offers a quality protein source with potential supplement and food production applications. Ongoing research aims to unveil its composition and explore its benefits across industries [24]. In a recent study by Uzlasir et al. [23], *Limospira* contained 17 amino acids, with alanine, aspartic acid, and glutamic acid being the most prevalent. *P. tricornutum*, on the other hand, was identified to have 19 amino acids, with glutamic acid being the most dominant. However, variations in amino acid quantities across studies were noted to be influenced by growing conditions [23,24]. Efforts have been made to leverage microalgal proteins’ foaming and emulsifying properties to enhance food sensory qualities. Studies have shown that adding Spirulina to chocolate [25] can improve its mouthfeel fragility, with a more pronounced effect observed with increased microalgal content. Similarly, Prandi et al. [26] explored using *Chlorella vulgaris*, *Tetraselmis chui*, and *Nannochloropsis oceanica* in a vegetable cream to maintain its texture while boosting nutritional value. Microalgal proteins have also been looked at as effective emulsifiers in food production; for instance, Rodrigues et al. [27] demonstrated that Spirulina extracts containing phycocyanin could replace traditional additives in ice cream, providing stabilizing properties without compromising consumer acceptability. Additionally, Almeida et al. [28] successfully created an instant sauce enriched with 4% Spirulina that remained stable in appearance and taste for up to 45 days. Fratelli et al. added Spirulina to bread, replacing 3% of wheat flour, and this substitution did not impact the rheological characteristics of the dough [29].

All these studies confirm the great potential of the use of microalgal macronutrients as ingredients in food. In addition to the macronutrients mentioned, the next section will discuss bioactive compounds and secondary metabolites known for their positive impact on health. These compounds affect physiological and cellular functions. Despite their often limited presence in foods, they offer substantial health advantages.

## 3. Microalgal Bioactive Compounds and Biological Effects

This section provides a concise and precise description of the experimental results, their interpretation, and the experimental conclusions that can be drawn regarding microalgal bioactive compounds and their consequent biological effects.

Bioactive compounds are natural dietary antioxidants that can be supplemented or applied in food matrices to prevent chronic diseases associated with Reactive Oxygen Species (ROS). Since these radicals have oxidation capabilities, which damage signaling proteins, lipids, cells, and DNA in the human body, generating endothelial injuries, cancer, and inflammation [30,31], the scavenging capacity of antioxidants is a crucial pathway to neutralize ROS. In this context, microalgae often offer a dual beneficial effect by scavenging free radicals, inhibiting lipid peroxidation in vivo, and enhancing the body’s endogenous enzymatic antioxidant mechanisms [32].

### 3.1. Hematological Parameters

Amalgamating the results that evaluate hematological parameters, we can cite the work conducted by Li et al. [33]; the authors showed that fucoxanthin chlorophyll proteins in *Phaeodactylum tricornutum* were enhanced by manipulating the expression of the gene violaxanthin de-epoxidase-like protein 1 (VDL1). This resulted in higher levels of fucoxanthin in microalgae and increased growth rates, advancing the potential for algae to serve as a viable commercial source for pigment production [33]. Through a general linear model after 6 and 12 weeks of follow-up testing, the daily supplementation of 37 women with fucoxanthin at 4.4 mg for 12 weeks showed a significant increase in bone mineral density while maintaining the bone mineral content for those during exercise and diet intervention. No effects were observed on the weight loss or blood pressure of the participants; however, the lipid profile was significantly affected by the fucoxanthin supplementation, with a decrease in low-density cholesterol (LDL) and very low-density cholesterol (VLDL) after 12 weeks [34].

A double-blind, randomized, placebo-controlled study with 104 volunteers supplemented a commercial ethanolic extract of *Nannochloropsis* sp. for 12 weeks [35]. The extracts were standardized to contain 250 mg of Eicosapentaenoic Acid (EPA), 150 mg of chlorophyll, 150 mg of lipids, 90 mg of palmitoleic acid, 40 mg of arachidonic acid (ARA), 23 mg of phytosterol, 764 µg lutein, 541 µg beta-carotene, and 387 µg zeaxanthin. The supplementation significantly increased the EPA and Docosapentaenoic Acid (DHA) in blood erythrocytes. Both subgroups, with and without high-cholesterol individuals, had significant decreases in the VLDL and, consequently, in total cholesterol after 12 weeks of intervention. It is worth mentioning in this section that hip circumference and body weight were significantly decreased by the microalgal extract supplementation.

Biotechnological applications in daily life remarkably changed the blood lipid profile and anthropometric measures through microalgae supplementation. These effects have been associated with cardiovascular protection and improved quality of life [35,36,37]. 

### 3.2. Antidiabetic Effects

Microalgae are a valuable resource for discovering metabolites with biotechnological significance, notably including glucosidase inhibitors for diabetes adjuvant treatments. A 5:1 lutein–zeaxanthin ratio extracted from *Chlorella* sp. attenuated atherogenesis and improved glucose homeostasis in diabetic ApoE−/− mice [38]. The authors found that the microalgal extract significantly reduced glucose tolerance by 25% and insulin resistance by 41%, enhanced biochemical indices related to blood glucose metabolism, and lowered inflammatory molecule expression. They restored antioxidant enzyme SOD, CAT, and GSH-Px activities with no significant differences between the *Chlorella* sp. extract and the controls in the eyes, heart, liver, and kidneys of mice. Additionally, reductions in liver lipid accumulation and improvements in thinning the outer nuclear layer (ONL) in the eyes were observed. An in vitro and in silico study [39] applied terpenoids (isoglabrolide and armillarin), alkaloids (Lucidine B and lappaconitine), and fatty acids (6-Hydroxypentadecanedioic acid and (9Z,11E,13E,15Z)-4-Oxo-9,11,13,15-octadecatetraenoic acid) from *Chlorella minutissima*. Lucidine B, isolated from the purified extract fractions, interacted with the target protein 3A4A with the lowest binding energy and formed a hydrogen bond interaction with the amino acid Glu408, indicating that it inhibits glucosidase (IC50 value of 1.61 mg.mL^−1^) through a non-competitive mechanism. α-glucosidase inhibition plays a vital role in carbohydrate metabolism and postprandial glycemic control, ameliorating diabetic conditions [40]. 

### 3.3. Neuroprotective Effects 

Carotenoids from *Nannochloropsis oceanica* and *Tisochrysis lutea* obtained through pressurized liquid extraction (PLE) presented moderate and selective inhibitory potential against acetylcholinesterase (AChE) and butyrylcholinesterase (BchE) enzymes, which play a role in the treatment of Alzheimer’s disease [41]. *N. oceanica* mainly had violaxanthin, antheraxanthin, and vaucheriaxanthin, derivatives, and esters from these xanthophylls at a concentration of 115.1 ± 0.6 mg.g^−1^. At the same time, the *T. lutea* extract presented isomers of fucoxanthin, mostly its all-*trans* isomer, at a concentration of 122.4 ± 4.7 mg.g^−1^. The *T. lutea* extract resulted in anti-AchE activity (IC_50_ = 47.17 μg.mL^−1^) and so did the *N. oceanica* extract (IC_50_ = 66.29 μg.mL^−1^). However, these values were significantly lower than the positive control (the widely used drug galantamine) and presented moderate inhibitory effects. The extract from *N. oceanica* exhibited the highest antioxidant activity (ABTS) of IC_50_ = 10.00 μg.mL^−1^ and that from *T. lutea* the highest anti-inflammatory activity (LOX) of IC_50_ = 28.45 μg.mL^−1^ after the controls, Trolox and quercetin, respectively. The results showed that none of the microalgal extracts exhibited significant cytotoxicity at concentrations of 25 and 50 μg.mL^−1^, and the *N. oceanica* extract displayed the greatest suppression of pro-inflammatory cytokine release, suggesting that this microalgal extract could be the most promising candidate for neuroprotection.

Comparing the thermal degradation preservation of Human Red Blood Cells (HRBCs), *P. tricornutum* had around 32% HBRC protection, and *Tetraselmis* sp. had approximately 18% [42]. Both species presented an average 38% inhibition of the cyclooxygenase-2 enzyme, thus providing neuroprotection against neurodegenerative diseases. The authors discussed the presence of fucoxanthin, ômega-3, and polysaccharides of *P. tricornutum* as responsible for the great anti-inflammatory activity.

## 4. Processing Techniques to Obtain Microalgal Ingredients for Food Application

Microalgae are considered raw materials rich in substances that can be added as food ingredients, such as natural pigments (carotenoids, phenolic compounds, phycobiliproteins, and chlorophyll), as well as macronutrients such as proteins and polyunsaturated fatty acids (PUFAs) like omega-3 and omega-6. The production of microalgae allows for high biomass cultivation and the consequent accumulation of bioactive compounds [43]. The extraction and purification processes of these high-commercial-value compounds constitute a significant part of the production process related to microalgal cultivation. Extraction methods should be tailored to the type of microalgae and the desired isolated product, aiming at preserving the chemical structure and maintaining the biological activity of the compounds [44].

Microalgae have multilayered cell walls that act as a barrier to the passage of solvents into the intracellular medium where the compounds of interest are concentrated. Therefore, an ideal cell wall disruption treatment is one that selectively releases the target product while using minimal energy. The choice of cell disruption method depends on the cell wall structure of the microalgal species, product location, size, polarity, and solubility. Disruption processes can be classified as physical, mechanical, chemical, or biological [45,46].

Several factors must be considered when choosing processing techniques to obtain microalgal ingredients for food applications to ensure optimal extraction efficiency and product quality. Here are some key points to keep in mind when selecting processing techniques, including the target compounds, extraction efficiency, scalability, cost, environmental impact (the sustainability of the processing technique, considering factors such as solvent toxicity, waste generation, energy consumption, and overall environmental footprint), selectivity and specificity, safety and regulations, and compatibility with food applications (how well the extracted microalgal ingredients will integrate into the intended food products in terms of flavor, texture, stability, and shelf life, and also choosing a processing technique that preserves the quality and functionality of the extracted ingredients). By carefully evaluating these factors and considerations, it is possible to select the most appropriate processing technique to obtain microalgal ingredients for food applications that best align with one’s production goals, quality requirements, and sustainability objectives. From the literature, the processing techniques that must meet these criteria are considered in this section. A summary of the methods is presented in Table 1.

Pulsed electric field (PEF) and sonication (PEF) are physical methods of cell disruption. PEF is a non-thermal method of cell permeabilization that ruptures the cell membrane’s lipid bilayer, allowing the passage of low-molecular-weight molecules into the intracellular medium. In different PEF treatments, sonication permeabilizes the cell membrane and the cell wall using ultrasound waves based on bubble cavitation [46,64,65,66].

Mechanical cell disruption methods are the most used as they do not cause significant damage to the compounds of interest. Bead milling is a high-intensity cell disruption method caused by high-speed spinning steel, zirconium, glass, or ceramic beads colliding with microalgal cells. This method is often used on an industrial scale as it exhibits high disruption efficiency and allows for operation with high biomass density. High-pressure homogenization is another mechanical disruption method in which the biomass is forced, under pressure, to pass through a narrow opening. This method induces rapid cell disruption even in organisms with highly resistant cell walls [67,68,69,70].

Enzymatic treatment for cell disruption generally results in more efficient product extraction than mechanical or chemical methods (using acids or bases that can denature proteins). Success in using enzymes depends on the composition and complexity of the specific microalgal cell wall. Often, the disruption process depends on applying more than one type of enzyme to break down particular molecules of the cell wall. However, enzymatic treatment requires mild pressure and temperature conditions, which implies an energy-efficient, non-hazardous, and environmentally friendly alternative compared to other methods [71,72].

These methods are often combined to optimize the extraction process. The main obstacle in the cell wall disruption process lies in controlling the damage caused, which can extend to other molecules of interest, especially proteins. One possible way to solve this problem is to combine disruption methods to use milder conditions during operation and increase extraction efficiency [71,73,74].

After the cell wall disruption process, refining and purification operations must be applied. Various unit operations are employed to optimally extract the compound of interest. Solid–liquid extraction (SLE) is the most frequently reported technique in the literature, typically using organic solvents applied directly to the biomass. The most used solvents are methanol, ethanol, acetone, n-hexane, and their mixtures, either separately or in distinct stages. However, conventional SLE uses a large amount of solvent, generating toxic residues, posing risks of chemical transformation of the extracts and creating challenges in completely removing the solvent from the purified product. Furthermore, traditional SLE methods are characterized by limited efficiency [75].

For these reasons, new processes that reduce or eliminate toxic solvents and improve efficiency and sustainability are necessary. Based on green chemistry principles, the green extraction of natural products focuses on reducing energy consumption and unit operations, minimizing the use of petroleum-derived solvents, and delivering a safe and quality extraction. Below are some techniques that align with the principles of green extraction.

### 4.1. Ultrasound-Assisted Extraction (UAE)

UAE uses ultrasonic energy and various solvents to extract compounds of interest from plant matrices efficiently. Ultrasound consists of mechanical waves with frequencies above 20 kHz, beyond the human audible range (20 Hz to 20 kHz). The propagation of these waves in the solvent creates regions of compression and rarefaction, displacing molecules from their original positions. As the intensity of the sound waves increases, these cycles become more intense, forming small bubbles (cavitation bubbles) due to low pressure in the rarefaction zones. These bubbles grow through coalescence and subsequently collapse in the compression zone, causing cell structure fragmentation due to collision [76].

Various parameters can affect the effectiveness of the UAE. Medium parameters are related to the space where ultrasonic waves propagate. Thus, the nature of the solvent is the most relevant variable in this parameter. Solvent polarity is important to achieve the correct solubility of the compound of interest: for extracting polar compounds such as carbohydrates, phenolic compounds, and various proteins, water may be the most suitable solvent. However, non-polar solvents like hexane or chloroform extract non-polar compounds such as lipids and carotenoids, which do not follow green chemistry principles [77]. To address this issue, green solvents such as ionic liquids and eutectic solvents are being studied, which will be discussed later. Parameters such as viscosity and surface tension should also be considered as they affect cavitation [78,79].

Some parameters related to the type of microalgae also influence the extraction of the target compound, such as structure, pre-treatment (cell disruption processes), particle size (as reducing particle size increases contact surface and consequently the recovery rate of the product), and the solid–liquid ratio (as dry biomass improves solvent permeability and mass transfer). Physical parameters are related to the ultrasonic waves, such as power, intensity, and frequency, and to equipment metrics, such as extraction time and the shape and size of ultrasonic reactors (ultrasound bath and ultrasonic probe) [80].

These parameters affect cell disruption efficiency and the bioactive compounds’ release from the microalgae. Higher power and intensity levels can enhance extraction by promoting better contact between the solvent and microalgal cells, improving compound recovery rates [9,78,80]. Concerning extraction time, the duration for which the ultrasonic waves are applied can impact extraction efficiency. Longer extraction times may result in higher compound yields, as they allow more time for the bioactive compounds to be released from the microalgal cells. Additionally, the design and dimensions of the ultrasonic reactors, such as ultrasound baths or ultrasonic probes, can influence the distribution of ultrasonic waves and the uniformity of treatment. Optimal reactor geometry can ensure the proper exposure of the microalgae to ultrasonic waves, improving the extraction process. Overall, by carefully controlling these physical parameters and equipment metrics during the extraction process, researchers can optimize the efficiency of bioactive compound extraction from microalgae, leading to higher yields and the better quality of the final product [9,79].

### 4.2. Microwave-Assisted Extraction (MAE)

Microwaves are a type of non-ionizing electromagnetic radiation with frequencies between 300 MHz and 300 GHz. This process has been widely used to extract bioactive compounds from microalgae, such as carotenoids, fatty acids, polyphenols, and polysaccharides. Compared to traditional extraction processes, MAE offers higher thermal efficiency and better stability of thermally stable components present in the matrix while consuming less solvent and energy, which makes it highly suitable for industrial applications [81].

Microwave heating uses radiation energy to generate heat through two simultaneous mechanisms: ionic conduction and dipole rotation. Ionic conduction refers to the migration of ions under the influence of the electric field produced by microwaves, causing friction between the ions and the medium, generating heat. Dipole rotation occurs when dipolar molecules, under the influence of an electric field, are oriented toward the field and disoriented when the field is removed, as it occurs with microwaves. This rotation leads to collisions between these molecules, generating heat. In MAE, heat transfer is uniform throughout the medium, causing moisture evaporation from within the cells and a consequent increase in intracellular pressure. The pressure variation causes cell membrane rupture and accelerates solvent penetration, releasing the intracellular compounds of interest [57].

As with UAE, solvent-related parameters significantly influence MAE. Due to their high natural moisture content, microalgae are susceptible to microwave processes, facilitating the release of target compounds. The solvent, however, must exhibit a high dielectric constant and strong energy absorption from the process. Thus, the solubility of the bioactive compound in the solvent and the ability to absorb microwave energy are crucial properties in solvent selection. Nevertheless, the stability of the target compound in response to temperature increases must be considered, as the application of MAE is limited in thermolabile substances [82].

### 4.3. Supercritical Fluid Extraction (SFE)

Although reduced, the use of non-polar solvents in extraction processes can cause issues related to residues in the final extract, potentially hindering its application. Moreover, methods requiring increased temperatures may lead to the thermal decomposition of biomass components. SFE, however, allows for the preservation of the natural qualities of bioactive compounds, reducing the environmental impact of chemical residue generation and minimizing the energy demand of the process [83].

SFE can be defined as a process in which a substance (typically CO_2_) reaches a critical point (specific temperature and pressure) where the liquid and gas phases coexist. The supercritical fluid is injected into the biomass under appropriate temperature and pressure, thereby solubilizing the desired chemical compounds. Subsequently, the solvent and compounds are extracted from the extraction vessel, leading to an increase in temperature and a decrease in pressure, causing the solvent to return to its gaseous state and leaving the target compound free of solvent [84].

SFE is particularly suited for extracting non-polar compounds, especially lipids and carotenoids, as they yield higher extraction rates. For the effective extraction of polar compounds, the concurrent use of a solvent with the supercritical fluid, such as methanol, is necessary. Using CO_2_ as a supercritical fluid is advantageous due to its easy availability, low cost, non-toxicity, low critical temperature (31.1 °C), and recyclability [85].

The limitations of SFE may include the low solubility of water-soluble compounds in CO_2_, though using co-solvents addresses this selectivity issue. Additionally, the high cost of implementation is a concern; however, the high yield, the purity of the obtained extracts, and the suitability for large-scale use mitigate this limitation [86].

### 4.4. Pressurized Liquid Extraction (PLE)

PLE uses high system temperatures and high pressure to maintain the solvent in a liquid state for compound extraction. The adjustment of temperature and pressure is related to the type of solvent used. The biomass is placed in an extraction tank where the solvent is injected, and due to the high temperature and pressure, compounds are extracted. This condition allows for reduced solvent viscosity and increased diffusion rate, significantly shortening the extraction time compared to other techniques. After extraction, the solvent is purged with nitrogen (N_2_). PLE offers advantages such as rapid extraction, small solvent volumes, generally automated and scalable processes, and higher extraction yields [87].

This process can target both polar and non-polar compounds depending on the solvent used. Following green chemistry guidelines, solvents like ethanol and water can be used in the process, depending on the target compound. Ethanol as an extraction solvent is widely reported in the literature for extracting lipids, carotenoids, and chlorophyll [86]. The operating temperature can range from 20 °C to 200 °C, allowing adjustment based on the sensitivity of compounds to temperature increases [88]. The main disadvantage of this extraction technique is the high implementation cost required for operation due to the high-pressure process [86].

Although microalgal biomass cultivation has grown in recent years, the commercialization of isolated compounds has yet to reach its peak. This is typically attributed to the high costs associated with the extraction and purification processes. Thus, economically viable methods for extraction and purification are necessary to enhance the cultivation of microalgae and the commercialization of their bioactive derivatives for food purposes.

All these processing techniques also have disadvantages, such as high equipment costs, an energy-intensive nature, and limited scale-up potential; considering, for example, UAE, scaling up for commercial production can be challenging due to equipment limitations and scalability issues. Similarly, we can cite downsides such as uneven heating and limited penetration depth for MAE [89]. Regarding SFE, high operating pressures are often a concern. For PLE, extraction efficiency will depend on the choice of solvent, and operating conditions in pressurized liquid extraction can significantly influence extraction efficiency and selectivity [9]. Moreover, there are also sample size limitations since pressurized liquid extraction may have limitations in processing larger sample volumes efficiently, restricting its applicability for industrial-scale production [90]. While these processing techniques offer unique advantages in extracting bioactive compounds from microalgae, it is essential to consider these disadvantages and challenges to optimize their application in food production and address potential limitations for commercial implementation [88,90,91,92].

## 5. Challenges, Gaps of Knowledge, and Future Prospects

Microalgal ingredients are creating waves in the food industry. These tiny powerhouses are packed with proteins and an outstanding amino acid profile, setting the stage for a protein transition due to its nourishment, greener alternative, and versatility [2,93]. Proteins are suitable for various food applications, from effective gelling and emulsifying properties to effective foaming [94,95,96]. Texture manufacturing according to individual preferences, such as veganism and vegetarian diets, while promoting a healthier lifestyle will evolve consumers’ food choices even more. Taste and color preferences pose challenges in catering to consumer needs, but innovative strategies have been studied, from cleverly masking flavors to exploring new fermentation techniques; microalgal ingredients promise the creation of diverse flavor profiles [11,38,39,40].

Moreover, the natural pigments provided by microalgal sources bring many health benefits since they offer a punch with antioxidant properties [8] for preventing inflammation and neurodegenerative diseases, improving diabetic responses, and boosting hematological markers [35,39,63], but maintaining their stability during and after the extraction is mandatory. It is worth mentioning that the physical or chemical extraction process discussed here can act as a non-selective method for target compounds, resulting in extracts that contain a mixture of bioactive compounds. The mixture could have synergistically novel effects on human health. Thus, this interaction must be addressed explicitly in the scientific literature.

The practical applications of bioactive compounds from microalgae in food are vast and promising. Some possibilities include nutritional enrichment since these compounds can be incorporated into various food products to enhance their nutritional profile. Bioactive compounds derived from microalgae possess antioxidant, anti-inflammatory, and other health-promoting properties [97]. They can be used to develop functional foods and dietary supplements targeted towards specific health concerns. Certain pigments extracted from microalgae, such as astaxanthin and C-phycocyanin, can be used as natural food colorants, as already mentioned [29,98]. With the growing consumer demand for clean-label and natural products, these compounds offer a sustainable alternative to synthetic dyes. Moreover, some microalga-derived compounds have unique flavor profiles that can enhance the taste and aroma of food products. These compounds can be utilized to develop novel food formulations with distinctive sensory attributes [11,99,100]. Other aspects include texture and stability improvement since polysaccharides and proteins from microalgae can act as emulsifiers, thickeners, and stabilizers in food formulations [2]. These compounds are crucial in improving various food products’ texture, mouthfeel, and shelf stability. Overall, the diverse range of bioactive compounds in microalgae offers exciting opportunities for innovation in the food industry, leading to the development of healthier, more functional, and visually appealing food products.

Meanwhile, addressing the bioactive compounds’ stability over changes in pH and temperatures is imperative since food post-treatments frequently include these changes, such as dry heat baking, steaming, freeze-drying, and other methods. Thus, understanding their conjugation with ingredients in food formulations will improve their applicability, and further studies are necessary, as is an essay on their sensorial acceptance. Additionally, future steps encompass overcoming obstacles around large-scale production, environmental impact, and regulatory frameworks. It is time to invest in research, innovation, and collaboration to unlock the full potential of microalgal ingredients [99,101].

Future perspectives regarding microalgal ingredients for food applications paint a promising picture of innovation, sustainability, and health-conscious food products. Some key points to consider in the future development of microalgal ingredients for food applications include expanding functional ingredients and promoting sustainable food production since microalgal cultivation offers a sustainable and eco-friendly alternative to traditional food production methods. Future microalgal farming and processing efforts will likely maximize efficiency, reduce resource inputs, and minimize environmental impact to contribute to more sustainable food production systems [3].

Similarly, the unique properties of microalgal ingredients, including their flavor profiles, textures, and functionalities, open up opportunities to develop novel food products. Future innovations may lead to the creation of innovative food formulations, supplements, and functional foods that cater to changing consumer preferences and dietary trends [2,102]. It is also indispensable to improve the knowledge of the bioavailability and nutrient absorption of microalgal ingredients, and ongoing research in microalgal processing and formulation may focus on improving the bioavailability and nutrient absorption of microalga-derived ingredients in the human body. Enhanced delivery systems and formulations can optimize the uptake of bioactive compounds, enhancing their health-promoting effects [29,102]. 

Collaboration between food scientists, biotechnologists, and researchers across disciplines can drive further innovation in microalgal ingredients for food applications. Collaborative efforts may lead to discovering new microalgal species, extraction techniques, and product formulations that revolutionize the food industry.

In addition, when selecting processing techniques to produce high-quality products using microalgal bioactive compounds to make the food of the future, the target compounds, extraction efficiency, scalability, cost, environmental impact, selectivity and specificity, safety and regulations, and compatibility with food applications should be considered.

The journey toward unleashing the true power of microalgae ingredients is just beginning. With continued advancements, research, and a dash of creativity, the way to a brighter, healthier, and more sustainable future will be successfully opened.

## Figures and Tables

**Table 1:** Cell disruption methods and types of microalgae studied in recent years.

Method	Microalgae	Study Aim	Reference
Pulsed electric field	*Chlorella vulgaris*	Increase in digestibility	[47]
Pulsed electric field	*Auxenochlorella protothecoides*	Lipid yield	[48]
Pulsed electric field	*Chlorella zofingiensis*	Carotenoids	[49]
Pulsed electric field	*Chlorella* sp.	Retention of gelling capacity	[50]
Pulsed electric field	*Chlorella* sp.	Antioxidant biomolecules	[51]
Ultrasound-Assisted Extraction	*Chlorella* sp.	Lipid yield	[52]
Ultrasound-Assisted Extraction	*Limno* *spira platensis*	Bioactive pigments	[53]
Ultrasound-Assisted Extraction	*Nannochloropsis*	Lipid yield	[54]
Ultrasound-Assisted Extraction	*Limno* *spira platensis*	C-phycocyanin	[55]
Ultrasound-Assisted Extraction	*Porphyridium purpureum*	C-phycocyanin	[56]
Microwave-Assisted Extraction	*Chlorella vulgaris*	Chlorophyll, carotenoid, and phenolic compounds	[57]
Microwave-Assisted Extraction	*Nanochloropsis* sp.	Lipid and eicosapentaenoic acid	[58]
Supercritical Fluid Extraction	*Chlorella vulgaris*	Polyhydroxyalkanoate (PHA)	[59]
Supercritical Fluid Extraction	*Tetraselmis chuii*	Omega-3 fatty acids	[60]
Pressurized Liquid Extraction	Spirulina, *Chlorella* and *Phaedactylum tricornutum*	Antioxidant and anti-inflammatory compounds	[61]
Pressurized Liquid Extraction	Spirulina, *Chlorella* and *Phaeodactylum tricornutum*	Carotenoids and other bioactive compounds	[62]
Pressurized Liquid Extraction	*Tetraselmis chuii*	Bioactive compounds	[63]

## Data Availability

No new data were created or analyzed in this study. Data sharing is not applicable to this article.
